# Pioneering Insights into the Complexities of Salt-Sensitive Hypertension: Central Nervous System Mechanisms and Dietary Bioactive Compound Interventions

**DOI:** 10.3390/nu17243961

**Published:** 2025-12-18

**Authors:** Renjun Wang, Bo Xu, Xiping Liu, Qi Guo, Gregory Miodonski, Zhiying Shan, Dongshu Du, Qing-Hui Chen

**Affiliations:** 1 Department of Biotechnology, School of Life Science, Jilin Normal University, Siping 136000, China; rjw@jlnu.edu.cn (R.W.); 15567863193@163.com (B.X.); 15903495754@163.com (X.L.); 2 Department of Kinesiology and Integrative Physiology, Michigan Technological University, Houghton, MI 49931, USA; guoqi_hebphy@126.com (Q.G.); gjmiodon@mtu.edu (G.M.); zhiyings@mtu.edu (Z.S.); 3 Department of Physiology, Hebei Medical University, Shijiazhuang 050017, China; 4 School of Life Sciences, Shanghai University, Shanghai 200444, China

**Keywords:** salt-sensitive hypertension, high-salt diet, central nervous system, paraventricular nucleus, rostral ventrolateral medulla, bioactive compounds, dietary intervention

## Abstract

Salt-sensitive hypertension (SSH) is an important and common subtype of hypertension, whose pathogenesis involves multi-level regulation, including the central nervous system (CNS), metabolic stress, and epigenetics. Dietary bioactive compounds have emerged as a research hotspot for SSH intervention due to their safety and multi-target effects. Although existing studies have focused on the CNS regulation of SSH or the role of individual dietary components, there is a lack of comprehensive analysis integrating multiple mechanisms, systematically summarizing multiple compounds, and incorporating a clinical translation perspective. This review first outlines the mechanisms of CNS pathways, endoplasmic reticulum (ER) stress, mitochondrial dysfunction, and epigenetic modifications in SSH. Then, it systematically reviews the mechanisms of action and preclinical and clinical research progress of bioactive compounds, including capsaicin, taurine, gamma-aminobutyric acid, tea, and anthocyanins in SSH. In summary, this review systematically clarifies the complex regulatory network of SSH and the intervention potential of dietary bioactive compounds from an integrated perspective, innovatively proposes a precise dietary intervention framework, and fills the research gaps in the integration of multiple mechanisms and systematic evaluation of compounds in existing studies. This framework not only provides a new integrated perspective for the basic research of SSH but also offers key references for clinical dietary guidance, functional food development, and the formulation of targeted intervention strategies.

## 1. Introduction

Based on a pooled analysis of population-representative studies across 200 countries and territories, the number of adults aged 30–79 with hypertension worldwide doubled between 1990 and 2019, reaching 1.278 billion by 2019, with 626 million women and 652 million men affected. As a well-recognized key risk factor, hypertension is closely associated with various chronic diseases, including cardiovascular disease, cerebrovascular disease, and kidney disease [1]. As a clinically significant subtype of hypertension, salt-sensitive hypertension (SSH) is characterized by a marked salt intake-dependent elevation in arterial blood pressure (ABP). It accounts for 50% to 75% of hypertensive patients, who exhibit significantly higher risks of cardiovascular disease occurrence and mortality compared with salt-resistant hypertensive individuals [2,3,4]. SSH, as a special type of hypertension, is closely related to excess dietary salt intake. Limiting salt intake or using diuretics to reduce sodium levels in the body can significantly lower ABP. Vasdev et al. reported criteria for evaluating salt sensitivity, stating that if a person’s average ABP drops by 10 mmHg or more compared with the measured value at the end of the salt-loading phase after being given 2 L of physiological saline within 4 h, the person is classified as salt sensitive [5,6]. In contrast, individuals are classified as salt-resistant if their ABP decreases by less than 5 mm of mercury. Based on these criteria, researchers observed that 51% of hypertensive patients demonstrated salt sensitivity. Notably, even among people with normal ABP, 26% also exhibited this trait. Importantly, salt sensitivity and salt resistance follow a normal distribution in both hypertensive and normotensive populations [7].

As the core sensory and regulatory hub for systemic salt metabolism and ABP regulation, the mechanisms underlying the role of the central nervous system (CNS) in SSH have been increasingly elaborated in recent years. Studies have demonstrated that circumventricular organs (CVOs) exhibit high sensitivity to subtle changes in sodium chloride concentration and osmotic pressure in plasma or cerebrospinal fluid (CSF). Key CVOs involved in this process include the organum vasculosum of the lamina terminalis (OVLT) and subfornical organ (SFO), which are circular structures adjacent to the third ventricle of the brain. This sensitivity enables CVOs to directly sense internal environmental fluctuations induced by salt intake. These sensory signals further activate sympathetic nerve activity (SNA) and regulate vasopressin release, ultimately participating in the pathogenesis and progression of SSH by affecting peripheral vasoconstrictor function and renal sodium excretion efficiency [8]. Meanwhile, endoplasmic reticulum (ER) stress-induced abnormal protein folding, mitochondrial dysfunction-caused energy metabolism disorders, and the synergistic interaction between epigenetic modifications and CNS regulatory mechanisms collectively constitute the complex pathophysiological network of SSH [9,10,11]. This intricate network underlies the pathogenesis of SSH, presenting substantial challenges to the development of effective disease intervention strategies.

Currently, the clinical management of SSH still relies primarily on strict salt restriction and antihypertensive drug therapy. However, long-term salt restriction regimens are commonly plagued by poor patient compliance [12], and some patients may also develop drug resistance or face an increased risk of target organ damage. Therefore, there is an urgent need to explore safe, effective, and easy-to-adhere-to adjuvant intervention strategies. A recent randomized controlled trial involving 72 patients with essential hypertension provided key evidence for the feasibility of self-administered salt reduction. In the trial, the intervention group voluntarily reduced sodium intake through dietary guidance, with 24 h urinary sodium excretion decreasing by 66 mmol compared with the control group. After 4 weeks of intervention, the group’s 24 h ambulatory systolic ABP and diastolic ABP were reduced by 9 mmHg and 5 mmHg, respectively, and no serious adverse events were reported [13]. This result confirms that self-initiated salt reduction without strictly standardized meals can achieve clinically meaningful ABP reduction. Derived from natural food ingredients, dietary bioactive compounds possess unique advantages of high safety and broad target specificity, and their potential in SSH intervention has become a research hotspot [14]. In recent years, studies have found that catechins in tea and other compounds can exert hypotensive effects by regulating body metabolism and improving internal environmental homeostasis [15]. Moreover, γ-aminobutyric acid (GABA), taurine, and others can alleviate high-salt-intake-induced hypertension by affecting peripheral vascular homeostasis [16,17,18,19]. The underlying mechanisms of action of these compounds will be systematically elaborated in subsequent chapters.

In existing studies on SSH, most focus on a single pathological mechanism or analyze the role of a specific type of dietary component in isolation, lacking a systematic connection between the multi-level pathogenic logic of SSH and the intervention value of dietary bioactive compounds [20,21,22,23,24]. This review will connect the pathogenic mechanisms of SSH at the CNS, cellular stress, and epigenetic levels, along with the effects of dietary compounds such as capsaicin, taurine, and tea bioactive components. Its aim is to clarify how different compounds target specific pathological links in SSH. For example, GABA is more suitable for SSH driven by central neurotransmitter imbalance, while taurine exerts a significant intervention effect on SSH mediated by the interaction of multiple mechanisms. These mechanisms include peripheral target organ inflammation/oxidative stress, renin–angiotensin–aldosterone system activation, and ion channel dysfunction. This target specificity is the core basis for precise dietary intervention in SSH. Meanwhile, we pay attention to the daily accessibility and dose safety of the compounds, and particularly note that current evidence for the hypotensive effects of most compounds is mainly based on animal experiments. Only a few components, such as black tea (flavan-3-ols) and taurine, have sufficient human research support, and they can exert effects at daily dietary doses, showing higher clinical translation feasibility. In this way, this review not only systematically summarizes existing studies and provides references for subsequent research directions and practical dietary interventions, but also offers an integrated perspective for understanding the complex regulatory network of SSH and promoting the clinical implementation of dietary interventions.

## 2. The Regulatory Mechanism of CNS in the SSH

The CNS serves as the “core hub” for the abnormal regulation of ABP in SSH. By integrating peripheral high-salt signals, regulating SNA, and modulating the neuroendocrine network, it directly influences the pathogenesis and progression of SSH [25,26]. Compared with essential hypertension, the sensitivity of ABP to high salt in SSH patients is largely associated with the manner of central ABP regulation and related functional changes under high-salt conditions, and this central-level association is an important manifestation of the ABP characteristics of SSH [27].

### 2.1. CNS Autonomic Circuits and SSH

Circumventricular organs (CVOs), the anteroventral third ventricle region (AV3V), the hypothalamic paraventricular nucleus (PVN), and the rostral ventrolateral medulla (RVLM) are crucial brain regions that orchestrate the central regulation of SSH [28,29,30,31].

First, regarding the structural and functional characteristics of these key regions: the CNS is mostly enclosed by the blood–brain barrier, but certain areas—such as the choroid plexus, sections of the third and fourth ventricles, as well as CVOs including the subfornical organ (SFO) and the organum vasculosum of the lamina terminalis (OVLT)—lack this barrier and are classified as periventricular organs (PVOs) [32]. The capillaries in PVOs have membrane pores that allow macromolecules and polar substances to move from the bloodstream to surrounding tissues, enabling blood–brain substance exchange. Meanwhile, ependymal cells in PVOs interact with CSF and form connections with regions such as the SFO, OVLT, and area postrema (AP), functioning as “sensors” for peripheral signals. The AV3V region, which includes the periventricular area, hypothalamus, central part of the anterior optic nucleus, and extends into the anterior hypothalamus and PVN [33], has been a focus of animal studies: research on Dahl salt-sensitive (DSS) hypertensive rats showed that damaging the AV3V region reduces vasopressin secretion and lowers ABP, confirming its role in ABP regulation [34]. As a hub for integrating multiple physiological functions, the PVN consists of a midline parvocellular area and a dorsal magnocellular area—parvocellular neurons here have direct connections to the RVLM, nucleus of the solitary tract (NTS), and sympathetic preganglionic neurons in the intermediolateral nucleus (IML) of the spinal cord, and studies indicate that it regulates the pathological mechanisms of hypertension and heart failure [35,36,37]. The RVLM, in turn, is a core center for cardiovascular sympathetic regulation: it receives afferent inputs from peripheral sensory sources and central nuclei (e.g., the caudal ventrolateral medulla, raphe nuclei) and activates spinal sympathetic preganglionic neurons via descending axons, directly driving SNA [38].

**Figure 1:**
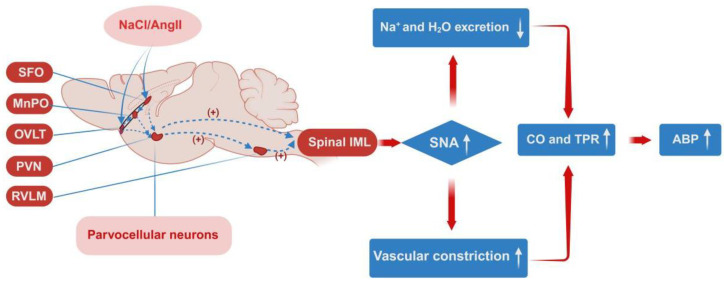
Autonomic pathways in the central forebrain that modulate SNA in SSH [40,43,44,45].SFO (Subfornical organ); MnPO (Median preoptic nucleus); OVLT (Organum vasculosum of the lamina terminalis); PVN (Paraventricular nucleus of the hypothalamus); RVLM (Rostral ventrolateral medulla); NaCl/AngII (Sodium chloride/Angiotensin II); Spinal IML (Spinal intermediolateral column); SNA (Sympathetic nerve activity); CO (Cardiac output); TRP (Transient receptor potential); ABP (Arterial blood pressure); Na (Sodium); H2O (Water). In this figure: Solid arrows indicate promoting effects; dashed arrows denote projections between central nuclei; and the “+” symbol represents functional promoting effects. Created in BioRender Renjun Wang, Bo Xu (2025), https://BioRender.com/t3gwjq5.

These brain regions work together through specific pathways to regulate SNA in SSH, as illustrated in Figure 1. The forebrain pathway that modulates central sympathetic nerve excitability is particularly fundamental: due to the incomplete blood–brain barrier in the SFO and OVLT, these regions can detect peripheral stimuli such as hypertonic salt and angiotensin II (AngII) [26,39]. Axons from SFO and OVLT neurons transmit signals to the median preoptic nucleus (MnPO), which then relays the stimulation to the PVN. Within the PVN, magnocellular neurosecretory neurons respond by sending signals to the posterior pituitary, triggering the release of arginine vasopressin (AVP) into the bloodstream—AVP further promotes sodium and water retention to elevate ABP. Additionally, PVN neurons that form monosynaptic connections with the RVLM (PVN-RVLM neurons) are responsible for sympathetic nerve regulation [40,41]. Both PVN and PVN-RVLM neurons receive inputs from the SFO and OVLT, and transmit this stimulation to spinal intermediolateral column (IML) sympathetic preganglionic neurons, ultimately enhancing peripheral SNA, inducing vasoconstriction, altering neurotransmitter release, and increasing ABP [42,43]. Notably, high salt intake and AngII exert a synergistic effect on the SFO-PVN-RVLM and OVLT-PVN-RVLM pathways, further amplifying sympathetic overactivity and increasing ABP [40].

Moreover, central autonomic regulation among SSH exhibits a salt load-dependent characteristic: arterial baroafferent signals. For example, play an important role in body sodium balance regulation: under a normal-salt diet, sinoaortic denervated (SAD) Dahl salt-sensitive rats exhibit suppressed urinary sodium excretion, sodium retention, and significant elevations in ABP—changes not observed in salt-resistant rats. However, a high-salt load masks this baroafferent signal-dependent regulation of sodium excretion [46], highlighting that the role of the central autonomic pathway in SSH is closely linked to the level of salt diet intake.

### 2.2. Factors Leading to Increased PVN Neuronal Activity and Sympathetic Outflow in Patients with SSH

The following table summarizes factors that increased PVN neuronal activity and sympathetic outflow in SSH. As shown in Table 1, these factors collectively promote an increase in SNA, leading to elevated ABP and playing a role in the pathogenesis and progression of SSH.

#### 2.2.1. Ion Channels: SK Channels Expressed in the PVN and Excitability of Autonomic PVN Neurons

Small conductance Ca^2+^-activated K^+^ channels (SK channels) are core molecules that regulate the excitability of PVN neurons with axons projecting to the RVLM (PVN-RVLM). Their function is synergistically inhibited by high salt and AngII, directly leading to increased neuronal excitability and SNA (Table 1) [60]. Under physiological conditions, activating SK channels via an increase in calcium influx following action potentials mediates potassium efflux to inhibit their own overexcitation, thereby preventing excessive sympathetic activation and normalizing ABP. However, in SSH, the synergistic effect of a high-salt diet and AngII disrupts this balance [48,61], reducing SK channel currents and downregulating the SK channels' function. Further studies have confirmed that in hypertension models induced by subcutaneous AngII infusion combined with a high-salt diet, impaired SK channel function in the PVN is a key driver of sympathetic excitation: microinjection of an SK channel blocker into the PVN of rats on a normal-salt diet significantly increases SNA and ABP. In contrast, these responses are significantly attenuated in rats with AngII-high salt treatment, suggesting pre-existing functional impairment of SK channels in the PVN. Notably, a high-salt diet alone is able to reduce these responses, while the effect of AngII infusion alone is not statistically significant [47], indicating that high salt plays a dominant role in the downregulation of SK channel function [41]. Its synergistic effect with AngII ultimately promotes the pathogenesis and progression of SSH by enhancing SNA.

It is worth noting that existing mechanism validations use rat models induced by AngII combined with high salt intake, and the short-term hypertension induced in these models differs from the pathological process of human SSH associated with long-term high dietary salt. Although the salt concentration in high-salt diets used in animal experiments (e.g., 2% NaCl) is lower than the extreme levels in some early studies (4% or 8% NaCl), it is still higher than the daily high salt intake in humans (approximately 1–2% NaCl), which may exaggerate the regulatory effect of SK channels. Furthermore, there is a lack of direct evidence for the functional status of SK channels in the central PVN of human SSH patients. The applicability of the central SK channel mechanism in humans still needs to be verified by clinical studies. These contradictions and limitations not only affect the comprehensive understanding of the pathological mechanisms of SSH but also pose challenges for the development of clinical intervention strategies targeting SK channels.

#### 2.2.2. Intercellular Communication: Brain-Derived EVs Mediate Inflammation and Oxidative Stress Activation in the PVN

In recent years, research has uncovered that brain-derived extracellular vesicles (EVs) from hypertensive DSS rats serve as novel mediators in SSH pathogenesis. When injected into normotensive Sprague Dawley rats, DSS EVs significantly upregulated the mRNA levels of pro-inflammatory cytokines (TNFα, IL1β), chemokines (CCL2, CCL5), and the chronic neuronal activity marker FOSL1 in the PVN and lamina terminalis (LT), which are key cardiovascular regulatory regions. Meanwhile, they induced neuronal mitochondrial reactive oxygen species (mtROS) elevation, which synergizes with neuroinflammation to amplify sympathetic activation, thereby promoting the development of SSH [62].

#### 2.2.3. Inflammation/Oxidative Stress: Multiple Pathways Amplify PVN Regional Imbalance and Neurotransmitter Dysregulation

A high-salt diet is a core trigger for inflammatory responses and oxidative stress imbalance in the PVN. Under physiological conditions, pro-inflammatory and anti-inflammatory factors, as well as the oxidative and antioxidant systems in the PVN, maintain a dynamic balance. The GABA inhibitory pathway and norepinephrine (NE) excitatory pathway mutually constrain each other, ensuring the stability of SNA. However, this balance is disrupted in pre-hypertensive or SSH models induced by an 8% NaCl diet [56]: high salt can directly upregulate the expression of toll-like receptor 4 (TLR4) in the PVN. By activating downstream signals of myeloid differentiation factor 88 (MyD88), two key pathological effects are induced. First, it promotes the nuclear translocation of NF-κB p65, which drives the release of pro-inflammatory cytokines such as interleukin-1β (IL-1β), interleukin-6 (IL-6), and tumor necrosis factor-α (TNF-α). Second, it upregulates the expression of NADPH oxidase 2/4 and reduces the production of copper/zinc superoxide dismutase, leading to the massive accumulation of reactive oxygen species (ROS).

Further studies have confirmed that NF-κB activation acts as a core hub, upregulating the expression of the NLRP3 inflammasome in the PVN, recruiting the infiltration of microglia and CD4^+^/CD8^+^ T cells, and inducing the expression of chemokines and adhesion molecules such as CCL2, CXCR3, and VCAM-1, thereby forming an inflammatory amplification loop [55]. As a key upstream trigger, TNF-α can dose-dependently induce PVN neurons to express inflammatory mediators such as IL-1β and CCL5 in DSS rats. Moreover, both its baseline level and stress response intensity are significantly higher than those in normotensive rats, suggesting that salt-sensitive individuals have an inherent high inflammatory reactivity [59]. The cross-talk between the aforementioned inflammatory and oxidative stress pathways ultimately leads to neurotransmitter imbalance in the PVN, directly driving renal sympathetic nerve hyperactivity.

Targeted intervention experiments in the PVN have further verified the pathogenicity of this mechanism [58]: in high-salt-induced models, microinjection of TLR4 blockers, NLRP3-specific inhibitors, or NF-κB inhibitors can significantly reduce the levels of inflammatory factors and ROS in the PVN, effectively lowering ABP and SNA. However, after terminating NLRP3 blockade, ABP and inflammatory indicators increase again, confirming that the sustained activation of inflammatory/oxidative stress pathways in the PVN is crucial for maintaining the hypertensive phenotype. The multi-pathway synergistically amplified functional disorders in the PVN, through the cascade reaction of inflammation activation, which enhanced oxidative stress and sympathetic excitation, constitute an important pathophysiological basis for a high-salt-induced SSH.

#### 2.2.4. Signal Molecule Regulation: Salt Load-Dependent Protective Effects of CO and H_2_S

Carbon monoxide (CO) and hydrogen sulfide (H_2_S) are important gas signaling molecules with anti-inflammatory and antioxidant activities. CO is produced by heme oxygenase-1 (HO-1) in the body, while H_2_S possesses both neuromodulatory and hypotensive effects. Both exert salt-load-dependent protective effects against high-salt-induced hypertension by regulating the pathophysiological changes in the PVN (Table 1). Relevant studies using male DSS rats as models have shown that exogenous supplementation or enhancement of endogenous CO and H_2_S can achieve protective effects: For CO, microinjection of the CO-releasing molecule CORM-2 into the bilateral PVN or upregulation of endogenous CO production significantly enhances the antioxidant capacity in the PVN, inhibits the expression of pro-oxidative factors and pro-inflammatory cytokines, reduces ROS accumulation, thereby lowering ABP and alleviating SSH [52]. In contrast, HO-1 inhibitors block endogenous CO production, exacerbating pathological damage and ABP elevation. For H_2_S, microinjection of H_2_S donors into the bilateral PVN or enhancement of endogenous H_2_S synthesis exerts effects through similar dual antioxidant and anti-inflammatory mechanisms, increasing the antioxidant capacity and anti-inflammatory cytokine expression in the PVN, directly regulating neurotransmitter balance, inhibiting SNA, and thus attenuating hypertensive responses. Conversely, H_2_S synthesis inhibitors suppress endogenous H_2_S production, which reversely exacerbates the progression of high-salt-induced SSH [53]. In summary, the protective effects of CO and H_2_S on SSH are consistent: both target and improve oxidative stress and inflammatory imbalance in the PVN by exogenous supplementation of donors or protection of endogenous synthetic pathways, inhibit excessive sympathetic activation, and ultimately alleviate SSH, providing a potential strategy for clinical intervention targeting gas signaling molecules in the PVN.

However, the short-term high salt load commonly used in animal experiments differs from the pathological process of long-term chronic high salt intake in humans. Additionally, the direct PVN microinjection of exogenous donors cannot be directly translated into clinical therapeutic approaches, and the blood–brain barrier penetration efficiency, targeting, and long-term safety of oral or intravenous administration have not been verified. These contradictions pose significant challenges for the development of clinical intervention strategies targeting gas signaling molecules.

#### 2.2.5. Glial Cell Regulation: Gαi2 Protein Inhibits PVN Microglial Activation to Alleviate SSH

Gαi2 protein is a key molecule regulating PVN function. It blocks high-salt-induced SSH progression by inhibiting microglial activation and subsequent inflammatory responses in the PVN, serving as a crucial central regulatory factor for maintaining ABP homeostasis under salt load (Table 1) [54]. Under normal conditions, PVN Gαi2 mediates sympathoinhibitory and ABP-stabilizing effects after high salt intake, preventing an increase in ABP. In salt-resistant rats, it regulates PVN neural activity to balance SNA and inflammation, ensuring adaptive ABP responses to high salt [54].

In the pathological process of SSH, Gαi2 dysfunction disrupts the normal regulatory balance. Using male Sprague Dawley rats as models, inhibition of Gαi2 expression induces significant SSH when combined with a high-salt diet. This pathological change is accompanied by upregulated mRNA levels of pro-inflammatory cytokines such as TNFα, downregulated mRNA levels of anti-inflammatory cytokines, and paraventricular nucleus (PVN)-specific microglial activation, confirming that Gαi2 deficiency triggers microglia-mediated inflammatory imbalance in the PVN and thereby drives SSH development [54]. In summary, this mechanism provides a novel therapeutic target: the Gαi2–microglia–PVN inflammation pathway for SSH.

#### 2.2.6. Neuropeptide Regulation: Orexin System-Mediated Sympathetic Activation and ABP Regulation

The orexin system, a key central neuropeptide regulatory system, plays a critical role in hypertension pathogenesis and is confirmed to participate in the pathological process of SSH (Table 1). High-salt load induces upregulated OX1R expression in the PVN: on one hand, it amplifies ABP regulation by enhancing arginine vasopressin (AVP) signaling; on the other hand, it activates the calmodulin-dependent kinase II (CaMKII) pathway, directly increasing SNA, leading increase in ABP. This effect is prominent in SSH models such as DSS rats but absent in salt-resistant rats, reflecting salt sensitivity characteristics [49,50,51].

Targeted intervention experiments verified the clinical value of this mechanism: microinjection of OX1R antagonists into the PVN of high-salt-fed DSS rats reduced MAP by −16 ± 5 mmHg, far superior to the −4 ± 4 mmHg in the normal-salt-diet group, effectively reversing high-salt-induced hypertension [49,50,51]. This indicates that PVN OX1R is a key node mediating SSH, and targeted inhibition of this receptor can block the orexin system’s pro-hypertensive effect, providing a novel direction and experimental basis for the precise treatment of SSH.

## 3. The Role of Subcellular Stress in SSH

Subcellular stress has received significant research attention regarding the complex pathological mechanism of SSH in recent years [10,63]. An increasing number of studies have shown that mitochondrial dysfunction and ER stress interact with neurons and glial cells in the core areas of ABP regulation, such as the hypothalamus and brain stem, jointly driving sympathetic overactivation and ABP dysregulation [64,65]. This subcellular damage is not only caused by oxidative stress under a high salt load but is also susceptible to genetic interactions, including the DSS hypertension animal model.

### 3.1. Mitochondrial Stress

Research has shown that a high salt intake, an important environmental trigger for SSH, can have multiple effects on the mitochondria of the CNS. Excessive salt intake can lead to changes in mitochondrial morphology, such as shortened mitochondrial length, reduced cristae count, increased mitochondrial fission, and intensified vacuolization [10]. A high salt load seriously damages the normal function of mitochondria. The oxidative phosphorylation process and electron transport chain of mitochondria are inhibited, resulting in a significant reduction in ATP production, which, in turn, affects the normal energy supply of neurons and interferes with the transmission of neural signals. The neural pathways related to ABP regulation are highly dependent on energy supply, and energy scarcity will inevitably affect their normal operation [66].

Excessive salt intake significantly increases mitochondrial oxidative stress levels. Mitochondria, as the main site for energy production within cells, experience increased electron leakage during electron transfer under high-salt stimulation, leading to a significant generation of ROS [67]. Not only do excessive ROS attack the membrane structures, proteins, and DNA of mitochondria, causing membrane lipid peroxidation, protein damage, and gene mutations, but ROS also further disrupt the normal function of mitochondria, forming a vicious cycle [68]. In summary, high salt intake significantly impairs cellular energy supply and signal transduction by inducing mitochondrial morphological abnormalities, disrupting normal mitochondrial function, and exacerbating oxidative stress, thereby providing an important pathophysiological basis for the occurrence and progression of SSH.

### 3.2. ER Stress

The ER, as an important organelle for protein synthesis, folding, and transportation, as well as calcium ion storage and regulation, plays a vital role in maintaining the normal function of neurons and glial cells. When the body is in a high-salt environment for a long time, the ER homeostasis in the CNS is easily disrupted, leading to ER stress.

Under normal circumstances, newly synthesized proteins fold correctly with the assistance of ER molecular chaperones. However, a high-salt environment can increase the demand for protein synthesis while disrupting the redox balance and calcium ion homeostasis within the ER, leading to the accumulation of large amounts of unfolded or misfolded proteins [9]. A chronic high salt intake also impacts the ER Ca^2+^ store function to modulate the PVN neuronal excitability. Studies found that a 2% NaCl high-salt diet augmented the excitability of PVN neurons projecting to the RVLM, and inhibiting ER Ca^2+^-ATPase to deplete Ca^2+^ stores contributes to this increased excitability, which may underlie the sympathoexcitation in chronic high salt intake [61]. Similar interactions between GRP78 and ER stress sensors and activation of the unfolded protein response (UPR) have been observed in many studies on diseases related to ER stress, such as diabetes and neurodegenerative diseases. For example, in a study of diabetes, a high-glucose environment contributed to ER stress, and GRP78 expression was upregulated and dissociated from its sensor, activating IRE1, PERK, and ATF6. These stress-induced transcription factors triggered the UPR to try to restore homeostasis to the ER. Although these studies did not directly target a high-salt diet and DSS rats, they provide strong supporting evidence for the mechanism of action of GRP78 under stress conditions. This indicates that GRP78 may also initiate UPR through a similar mechanism in ER stress induced by a high-salt diet [69].

## 4. The Pathogenic Mechanism of a High-Salt Diet Driving an Increase in ABP

The effect of a high-salt diet on ABP is not driven by a single mechanism but is achieved through integration of various mechanisms involving the CNS, immune system, vascular endothelium, endocrine system, and sex difference. The following provides a detailed analysis of its pathogenic mechanism from different perspectives.

### 4.1. Pathological Reactions of the Peripheral System to a High-Salt Diet

SSH is usually accompanied by increased sodium reabsorption in the distal renal units [70]. The peripheral RAAS also participates in the reabsorption of sodium in the distal renal units. Studies have shown that in salt-tolerant subjects, a high dietary salt intake can induce sodium stress and inhibit systemic RAAS. However, in salt-sensitive subjects, a high salt intake may promote sodium reabsorption independent of RAAS by directly activating mineralocorticoid receptors [71]. Therefore, a high-salt diet can stimulate the renin–angiotensin system, leading to vasoconstriction and elevated blood pressure.

### 4.2. A Salt-Rich Diet Can Lead to an Increase in Sodium Ion Concentration in CSF

Numerous studies have shown that a high-salt diet can lead to an increased sodium ion concentration in CSF. Using DSS rats and spontaneously hypertensive rats as models, it was found that when these rats were fed a high-salt diet, the sodium ion concentration in their CSF showed a significant upward trend, which is closely related to the occurrence and development of SSH. In contrast, rats with normal ABP did not show any changes in CSF sodium levels under a high-salt diet. This indicates that there are differences in the sensitivity of CSF sodium regulation mechanisms to a high salt intake between different strains of rats, and these differences are associated with susceptibility to hypertension [72,73,74,75]. The epithelial sodium channels (ENaCs) in the choroid plexus play a crucial role in the development of SSH. The choroid plexus is responsible for the generation of CSF, and a high-salt diet can upregulate the expression and activity of ENaCs in the choroid plexus. ENaCs help to transfer sodium ions from plasma to epithelial cells of the choroid plexus, which then enter the CSF through the sodium pump, ultimately leading to an increase in the total concentration of sodium ions in the CSF. This process clearly explains at the molecular level how a high-salt diet leads to an increase in CSF sodium concentration [76].

### 4.3. Immune Dysfunction Caused by High Sodium Intake

Studies have shown that excessive intake of salt in salt-sensitive individuals can have an impact on the immune system [77,78,79,80]. When mice consume too much salt, the production of Th17 cells can be induced by reducing the diversity of lactic acid bacteria, leading to hypertension in both mice and humans [81,82]. In addition, when exposed to high-salt conditions, initial T cells differentiate into various subgroups such as Th1, Th2, Treg, and cytotoxic CD8+T cells, which are influenced by the local microenvironment in different organs. Each T cell subpopulation may have significantly different effects on tissue damage and hypertension progression [83].

### 4.4. Deterioration of Endothelial Function Caused by High-Sodium Diet

Numerous studies have shown a link between endothelial dysfunction and SSH, highlighting its key role in potential pathological mechanisms. It is worth noting that when the biological activity of nitric oxide is reduced, the plasma level of the von Willebrand factor is increased, and urinary endothelin-1 excretion is significantly reduced [84]. As sodium levels increase, it leads to a decrease in nitric oxide release from endothelial cells [85]. In salt-sensitive hypertensive patients, the ability of nitric oxide to induce vasodilation is impaired, which presents prior to the onset of hypertension. As age and ABP increase, the release of nitric oxide decreases, worsening the disease progression.

### 4.5. Sex Dimorphism in SSH

Significant gender differences exist in the onset and progression of SSH, which has been confirmed by multiple clinical studies. In clinical practice, it has been observed that menopausal women, especially those who are genetically susceptible and experience estrogen deficiency and increased salt sensitivity, are at greater risk of developing hypertension, cardiovascular disease, and kidney disease. Related studies support sex differences regarding SSH. A high-salt diet increased the ABP of female mice without altering the ABP of males, and it has been found that a high-salt diet leads to an increase in adrenal mRNA expression of angiotensinogen and renin receptors in female mice [86]. We speculate that this is closely related to the differences in hormone secretion. Androgens affect the renin–angiotensin–aldosterone system (RAAS) in the kidneys, enhancing its activity, which in turn leads to further kidney damage and promotes the development of myocardial hypertrophy.

Women show greater sensitivity of aldosterone production to AngII. Under a high-salt diet, insufficient suppression of the RAAS results in sustained elevation of aldosterone levels, driving excessive activation of endothelial mineralocorticoid receptors (MR) [87]. Men predominantly rely on the activation of the AT1R (pro-vasoconstriction), while premenopausal women depend on the Ang (1–7)/MasR (angiotensin (1–7)/Mas receptor) pathway (anti-vasoconstriction). After menopause, this protective pathway weakens, shifting to a pro-hypertensive RAAS activation mode.

Studies have revealed gender-specific effects of T cells on SSH and renal injury. In DSS rats, depletion of functional T cells significantly alleviates salt-induced hypertension and proteinuria in both male and female rats, but female rats gain more pronounced protection against renal injury. Transplanting splenocytes from either female or male DSS rats into male rats lacking functional T cells exacerbates their hypertension and proteinuria. However, when transplanted into female rats of the same strain, only male splenocytes worsen the condition [88].

In summary, the sex dimorphism of SSH is supported by clear clinical and experimental evidence. Postmenopausal women have a higher risk of SSH and more specific pathological manifestations due to the activation characteristics of the RAAS and gender differences in T cell-mediated renal protection. In contrast, men are mainly characterized by the activation of the AT1R pathway and T cell-mediated pro-injury effects. This gender-specific divergence at the mechanistic level is a key driver of the onset and progression of SSH.

## 5. Epigenetic Modifications and SSH: Emerging Targets in Immunometabolism and Signaling Pathways

Epigenetic reprogramming, a key bridge linking environmental factors and genetic regulation, plays a central regulatory role in SSH. High salt intake induces epigenetic reprogramming in innate immune cells, endowing them with sustained responsiveness to repeated stimuli through transcriptomic and metabolomic remodeling. It also regulates T cell differentiation, forming an immune-mediated amplifying effect on ABP elevation [89].

### 5.1. Histone Methylation: A Regulator of Renal Sodium Metabolism in SSH

In the regulation of renal sodium metabolism, histone H3 lysine 4 trimethylation (H3K4me3) is enriched in the kidneys of deoxycorticosterone acetate (DOCA)-salt-induced hypertensive mice, while the expression of lysine-specific demethylase 5A (KDM5A) is downregulated, leading to excessive activation of the epithelial sodium channel (ENaC). Lithocholic acid (LCA) activates the G protein-coupled bile acid receptor (TGR5) and upregulates KDM5A expression via the JNK/c-Jun pathway, reversing H3K4me3 enrichment, inhibiting ENaC-mediated sodium reabsorption, and achieving ABP regulation. This finding reveals the critical role of histone methylation in renal sodium metabolism and provides experimental evidence for epigenetic targeted intervention [90].

### 5.2. Genetic Variation-Epigenetic Modification Interaction Mediates Salt Sensitivity

Genetic variation is a core determinant of individual salt-sensitive phenotypes. Specific gene polymorphisms confer disease susceptibility by altering molecular regulatory patterns. As a key component of the RAAS, the angiotensinogen (AGT) gene forms the hypertensive haplotype Hap-I and the normotensive haplotype Hap-II due to polymorphisms. Under a high-salt diet, the AGT gene promoter region in Hap-I carriers exhibits significant DNA demethylation, enhancing transcription factor binding capacity and resulting in significantly higher AGT expression than in Hap-II carriers. This clarifies the molecular mechanism by which the interaction between genetic variation and epigenetic modification mediates salt sensitivity [11].

### 5.3. Summary: Epigenetic Modifications as Core Mechanisms and Targets for SSH Intervention

In summary, epigenetic modifications regulate immune cell function, renal sodium metabolism, and the cross-synergy of multi-system signaling pathways. They serve as a core mechanism connecting high-salt environments to the occurrence and progression of SSH, and provide important directions for related targeted interventions.

## 6. Bioactive Compounds and SSH

**Figure 2:**
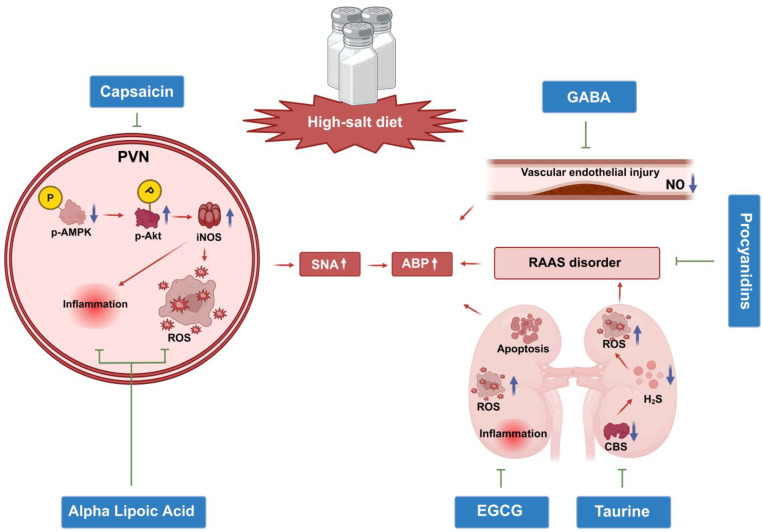
Schematic diagram of multi-target regulatory mechanisms of natural compounds towards hypertension induced by a high-salt diet. This figure illustrates the pathological process by which a high-salt diet induces hypertension: it activates oxidative stress and inflammation in the paraventricular nucleus (PVN) of the hypothalamus, increases sympathetic nerve activity (SNA), and causes renin–angiotensin–aldosterone system (RAAS) disorder, thereby leading to elevated arterial blood pressure (ABP). Among them, capsaicin acts on the PVN, inhibiting oxidative stress (ROS) and inflammation by regulating the phosphorylated Adenosine Monophosphate-Activated Protein Kinase (p-AMPK), Phosphorylated Protein Kinase-B (p-Akt), and Inducible Nitric Oxide Synthase (iNOS) pathways; Epigallocatechin Gallate (EGCG) improves renal oxidative stress, inflammation, and apoptosis; taurine acts on renal tissue, repairing and inhibiting renal tissue RAAS disorder through the cystathionine β-Synthase/hydrogen sulfide (CBS/H_2_S) pathway; γ-aminobutyric acid (GABA) improves vascular endothelial injury and increases nitric oxide (NO) levels; procyanidins specifically inhibit serum RAAS disorder. Ultimately, these natural compounds exert ABP-lowering regulatory effects through these multiple targets. Arrows: Blue upward arrows (↑) denote increased expression, activity, or level; blue downward arrows (↓) denote decreased expression, activity, or level; red arrows indicate promoting effects;⊥indicate inhibitory effects. Created in BioRender. Renjun Wang, Bo Xu (2025), https://BioRender.com/t3gwjq5.

Based on the core pathogenesis of central regulatory disorders in SSH described above, dietary bioactive compounds have been confirmed to exert interventional effects through multiple targets and pathways (Figure 2). Below is a systematic review of the functional characteristics, targeted regulatory mechanisms, dosage safety, and clinical translation potential of these compounds (Table 2).

### 6.1. Central Regulatory Mechanisms of Natural Bioactive Components: Novel Targets and Translational Potential for SSH Intervention

As natural bioactive components, capsaicin and α-lipoic acid (ALA) target central regulatory regions such as the hypothalamic PVN and modulate molecular signaling pathways including the AMPK/Akt/iNOS, RAAS and oxidative stress-inflammatory network. They provide a multi-targeted and pathway-specific intervention strategy for SSH, while their clinical translation requires attention to core issues such as dosage window, administration route, and species differences.

#### 6.1.1. Capsaicin: Central AMPK/Akt/iNOS Pathway Regulation and Bidirectional Effects of TRPV1 Receptors

As the core pungent active component of chili peppers, capsaicin exerts interventional effects on SSH mainly through three pathways: CNS regulation, renal protection, and transient receptor potential vanilloid 1 (TRPV1) activation (Table 2). At the central level, in experiments on DSS rats, capsaicin infusion into the PVN attenuated high-salt-induced hypertension, tachycardia, and myocardial hypertrophy. Its mechanism is associated with regulating the AMPK/Akt/inducible nitric oxide synthase (iNOS) pathway and balancing oxidative stress and inflammatory responses within the PVN (Figure 2). However, this administration route differs from human dietary intake, and the dosage lacks a reference value [91].

In terms of renal protection, low-dose capsaicin (1 mg/kg) exerts a beneficial effect in salt-sensitive hypertension (SSH) models induced by renal ischemia–reperfusion (I/R). It reverses renal injury and hypertension by activating TRPV1 receptors. In contrast, high-dose capsaicin (100 mg/kg) exacerbates these pathological changes, indicating a clear dose-dependent effect of capsaicin in this context. Activation of TRPV1 channels can protect renal tissue after renal I/R injury through anti-inflammatory and antioxidant stress effects, while preventing high-salt-diet-induced hypertension [92]. An epidemiological study involving 9273 volunteers also indicated that dietary capsaicin intake is associated with a reduced risk of hypertension [97]. However, there are significant species differences in salt sensitivity between animals and humans, and the translation of relevant mechanisms to the intervention of human SSH still lacks direct clinical evidence support.

There is an obvious contradiction in the interventional effects of capsaicin: central PVN infusion exerts an antihypertensive effect through the AMPK/Akt/iNOS pathway [91], while peripheral high-dose administration (100 mg/kg) exacerbates renal injury and hypertension [92]. This bidirectional effect may be related to target specificity: the central pathway focuses on anti-inflammation and antioxidant stress, whereas high peripheral doses may excessively activate TRPV1 receptors. As a non-selective cation channel, TRPV1 overactivation can disrupt its normal function of regulating cation influx, resulting in impaired ion transport balance that contributes to the exacerbation of pathological changes. This suggests that the interventional value of capsaicin is highly dependent on the administration route and dosage window, and whether its dietary intake form can achieve central targeting remains unclear. More cross-species studies are needed to clarify this contradiction.

Notably, although there are currently no human studies on capsaicin for SSH treatment, in clinical studies of diabetic peripheral neuropathy (DPN), the 8% capsaicin patch (Qutenza) has been confirmed to relieve pain and repair nerve function through promoting nerve regeneration, with sustained effects [98]. This administration route avoids the limitations of oral or central infusion, providing a feasible idea for subsequent clinical studies in SSH populations. In the future, the administration mode of this patch can be referenced to explore the efficacy and safety of capsaicin in intervening SSH by locally activating TRPV1 receptors and regulating related pathways, thus offering a new translational direction for the targeted treatment of SSH.

#### 6.1.2. Alpha Lipoic Acid (ALA): Central Oxidative Stress Inhibition and Regulation of the RAAS/Inflammatory Cytokine Network

Alpha lipoic acid is a natural antioxidant with both lipophilic and hydrophilic properties. Its core intervention target for high-salt-induced hypertension focuses on the hypothalamic PVN, a central regulatory site, which improves the central pathological microenvironment through multi-pathway synergy, thereby reducing SNA and ABP. In Wistar rat experiments, a high-salt diet (8.0% NaCl for 8 weeks) significantly increased SNA and ABP. It also induced oxidative stress imbalance, activation of the RAAS, and disorder of inflammatory factors in the PVN. Specifically, it upregulated the levels of superoxide, NAD(P)H oxidase subunits (gp91phox, gp47phox), angiotensin-converting enzyme (ACE), AT1R, and pro-inflammatory cytokines (IL-1β, IL-6), while decreasing the levels of the anti-inflammatory cytokine IL-10 and copper/zinc superoxide dismutase [57]. Supplementation with ALA via gastric perfusion (60 mg/kg for 9 weeks) significantly reversed the above pathological changes. It not only effectively inhibited oxidative stress in the PVN and downregulated the mRNA and protein expressions of gp91phox and gp47phox, but also suppressed excessive RAAS activation, restored the balance between pro-inflammatory and anti-inflammatory factors, and ultimately achieved a significant reduction in ABP and SNA [57].

In terms of mechanism, ALA is characterized by “central targeting + multi-pathway interaction”. ALA’s antihypertensive effect is not regulated by a single target. Instead, it relies on the synergistic effects of three key aspects: enhancing superoxide inhibitory capacity, inhibiting RAAS activation, and restoring the balance of inflammatory factors. These three aspects together form the core mechanism network through which ALA intervenes in high-salt-induced hypertension. Currently, this mechanism has been clearly verified in animal experiments, but there are still gaps in translational research. On one hand, the 60 mg/kg gastric perfusion dose in animal experiments needs to be converted into a human equivalent dose based on body surface area to evaluate the feasibility of daily dietary supplementation or health product intervention. On the other hand, there is no direct clinical evidence to support issues such as whether ALA has different intervention effects on salt-sensitive and salt-resistant populations, and the safety of long-term supplementation. Future studies should conduct targeted human clinical trials to clarify the optimal intervention dose, applicable scenarios, and long-term safety of ALA in SSH populations, as well as exploring the practical intervention value of dietary sources of ALA (such as spinach, broccoli, animal livers, etc.) to provide support for its translation from basic research to clinical practice [99].

### 6.2. GABA: Neurotransmitter Balance Restoration and Peripheral Vascular Homeostasis Regulation

GABA is a non-protein amino acid naturally present in the human brain, animals, plants, and microorganisms. It is formed by removing the carboxyl group of glutamate in the brain through the action of glutamate decarboxylase [100]. GABA is considered to be one of the inhibitory neurotransmitters involved in various metabolic activities, such as hypertension, diabetes, cancerous activity, oxidation, and inflammation [101].

In the central pathological process of SSH, a high-salt diet drives the activation of the NLRP3-TNF-α-ROS axis in the PVN, disrupting the balance between the excitatory neurotransmitter NE and the inhibitory neurotransmitter GABA. It also triggers peripheral pathological responses, ultimately exacerbating hypertension. In hypertensive mice induced by a high-salt and high-cholesterol diet, fermented GABA salt inhibited M1 macrophage polarization, alleviated endothelial dysfunction and vascular smooth muscle cell proliferation, and ultimately reduced ABP (Figure 2). The intervention dose of GABA salt in this experiment was set based on the daily dietary salt intake ratio of mice, which converts to a human equivalent dose of approximately 0.3–1 g per day. This dose can be achieved through daily intake of fermented foods without additional safety risks [94].

In a spontaneously hypertensive rat model, the GABA-rich tomato variety “DG03-9” significantly reduced ABP after a single administration and 4 weeks of chronic feeding. Moreover, the chronic intervention showed a better inhibitory effect on ABP elevation than ordinary tomatoes [95]. The dose corresponded to rats ingesting approximately 10–50 mg/kg of GABA through feed daily, converting to a human daily intake of about 0.5–2 g. This is consistent with the GABA content of natural foods such as fermented soybean products and specially cultivated fruits and vegetables (0.1–1 g/100 g), indicating practical dietary feasibility (Table 2).

Clinical correlation evidence further supports its value: a study on patients with chronic obstructive pulmonary disease complicated by pulmonary hypertension showed that serum GABA levels are a protective factor for ABP regulation. Together with risk factors such as NE and endothelin-1 (ET-1), GABA participates in ABP regulation. The area under the receiver operating characteristic curve of the combined diagnosis involving these four factors reached 0.966. This confirms the key role of GABA in the ABP regulatory network. Additionally, the physiological level of serum GABA in this study is consistent with the in vivo concentration range in humans after daily dietary GABA intake, indirectly verifying the safety of dietary GABA supplementation [17].

In terms of dose safety and practicality, the intervention doses in the aforementioned animal experiments were all set based on the GABA content of natural dietary sources. Humans can achieve the equivalent intervention dose (0.5–2 g/d) by consuming 100 g of fermented soybean products or 120 g of GABA-fortified fermented foods daily. Existing studies have shown that no obvious adverse reactions were observed in adults supplementing with 1–5 g of GABA per day. Only a very small number of people may experience mild bloating and dizziness due to individual differences, which are mostly likely to occur when the dose far exceeds 2 g/d. This intervention dose is much lower than the risk threshold [96], indicating good clinical translation safety and dietary operability.

### 6.3. Peripheral Target Organ Protection: Focus on Local Pathological Reversal of RAAS and Oxidative Stress

This class of compounds targets the RAAS and subcellular stress (e.g., ER stress, oxidative stress) as core targets. They indirectly block the pathological progression of SSH by repairing the structure and function of peripheral target organs such as the kidneys and blood vessels, and most possess the natural advantage of being both medicinal and edible (Table 2).

#### 6.3.1. Procyanidins: RAAS Inhibition and Vascular Function Protection

Procyanidins are a general term for a large class of polyphenolic compounds widely present in plants. Their common feature is that they can produce anthocyanins when heated in acidic media, hence they are called anthocyanins [102]. Anthocyanins not only help restore skin elasticity but also assist in maintaining the normal function of joints; arteries; and other tissues, such as the heart.

In recent years, a study extracted procyanidins from hibiscus calyces. In high-salt-diet-induced hypertensive rat models, experimental groups treated with different doses of procyanidin and a positive control group were established. The results showed that procyanidins dose-dependently reduced the ABP and HR of hypertensive rats, and significantly decreased serum angiotensin-converting enzyme (ACE) activity and plasma aldosterone levels [16]. Their antihypertensive effect was comparable to that of antihypertensive drugs, confirming that procyanidins exert anti-SSH effects by inhibiting the RAAS (Figure 2). The procyanidin doses of 50, 100, and 200 mg/kg in this study referenced the conventional dose gradient design of similar plant polyphenol intervention experiments on hypertensive rats [16]. Although some converted doses in this experiment exceed the basic level of daily human dietary intake, they are not without practical reference value.

Human intervention studies have provided insights into feasible procyanidin intake levels, with relevant trials exploring specific dosages in clinical settings. For example, a randomized double-blind placebo-controlled trial involving elderly individuals with mild cognitive impairment (MCI) administered a daily supplementation of 320 mg grape seed procyanidin extract (GSPE) over 6 months, representing a typical dose range investigated in human procyanidin research [103]. The results of the high-dose group can provide a reference for the efficacy threshold in the development of functional health products. Meanwhile, as a common medicinal and edible raw material, hibiscus calyces have been proven to have high safety in daily consumption as tea, further indicating that the dose design of this experiment has both scientific validity and translational potential [104,105].

#### 6.3.2. Tea Active Components: Subcellular Stress Inhibition and Renal Function Protection

Tea is a popular beverage worldwide, and China has a long history of tea consumption, with a large population of tea drinkers. Previous studies have found a close relationship between drinking tea and hypertension [106]. In addition to caffeine, black tea contains a large amount of plant polyphenols and flavonoids. Drinking tea regularly is associated with lowering ABP and reducing the risk of hypertension in the elderly. Research conducted in the laboratory has shown that tea and its secondary compounds play an important role in alleviating smooth muscle contraction, promoting endothelial nitric oxide synthase activity, reducing vascular inflammation, inhibiting rennin activity, and combating intravascular oxidative stress [107]. Black tea may alleviate endothelial dysfunction caused by AngII in hypertensive rats by inhibiting oxidative stress and stress in the ER of blood vessel walls [108].

Notably, as a key member of the critical active component family in tea, epigallocatechin gallate (EGCG), the main active catechin in green tea which has undergone targeted verification in animal experiments regarding its regulatory effects and mechanisms on SSH. A study using DSS hypertensive rats as models showed that after 6 weeks of continuous oral EGCG administration, the rats not only exhibited a significant reduction in ABP but also an obvious improvement in renal function. This was manifested by decreased 24 h urinary protein levels, optimized creatinine clearance rate, and alleviated renal fibrosis, suggesting that the antihypertensive effect of EGCG may be indirectly mediated through renal protection and improvement of renal injury (Figure 2) [15]. Further mechanistic studies indicated that the renal protective effect of EGCG stems from its antioxidant activity and inhibition of inflammatory responses [15]. This research reveals that EGCG improves SSH through a synergistic pathway of antioxidation and anti-inflammation, providing a new molecular mechanism reference for the intervention of SSH with tea active components.

It should be noted that the aforementioned protective effects of EGCG have only been verified based on the Dahl rat model so far, and their applicability in humans requires cautious evaluation. For example, there are differences in the administration dose, metabolic absorption efficiency of EGCG between animal experiments and human dietary intake scenarios. Additionally, black tea and green tea differ in the composition and content ratio of active components. Whether the mechanism of EGCG can be directly extrapolated to black tea and its human intervention effects still lacks support from direct clinical evidence.

A systematic review and dose–response meta-analysis included 13 randomized controlled trials involving 22 study groups, and the results showed that supplementing with black tea significantly reduced systolic and diastolic ABP. However, non-linear analysis did not find an effect of the dosage or duration of black tea flavonoid supplementation on ABP [109]. However, due to issues such as methodological heterogeneity and small sample sizes in existing clinical studies, there are conflicting research results. More large-scale, long-term, and strictly controlled clinical trials are needed to clarify the specific efficacy, optimal dosage, and applicable population of black tea in SSH treatment.

### 6.4. Taurine: CBS/H_2_S Pathway Restoration and Multi-Target Synergistic Effects

As a semi-essential sulfur-containing amino acid in the human body, the core interventional value of taurine in SSH lies in its ability to simultaneously target the core pathological mechanisms. It not only reverses the inflammation/oxidative stress imbalance in peripheral target organs but also regulates the RAAS [18,19]. A complete evidence chain has been established through comparisons between salt-sensitive and salt-resistant models, as well as human clinical trials [93].

In animal experiments, a high-salt diet significantly downregulated the endogenous taurine content and the mRNA and protein expression of synthetases in the kidneys of DSS rats. By inhibiting the renal cystathionine β-synthase (CBS)/(H_2_S) pathway, it exacerbated RAAS activation and oxidative stress, ultimately leading to renal injury and hypertension (Figure 2). Supplementation with 2%~3% taurine (added to drinking water for 4~6 weeks) exerted effects through multiple mechanisms: first, activating the renal kallikrein-kinin system, increasing urinary kallikrein excretion and renal tissue gene expression, promoting urine output and sodium excretion, while reducing the heart weight/body weight ratio and proteinuria to achieve antihypertensive and cardiorenal protection [18]; second, upregulating the expression and activity of renal H_2_S synthase (CBS), decreasing the levels of renin, angiotensin II, aldosterone, and oxidative stress indicators in renal tissue, enhancing antioxidant capacity and antioxidant enzyme activity, and reversing renal ultrastructural damage [19].

It should be noted that the 2~3% drinking water supplementation dose in animal experiments converts to a human equivalent dose of approximately 10~15 g per day, which is far higher than the daily dietary intake of adults. This belongs to a supraphysiological dose, and its core value lies in clarifying the intervention mechanism rather than directly translating to human intervention programs.

At the clinical translation level, a randomized, double-blind, placebo-controlled trial involving 120 prehypertensive individuals provided key evidence: daily supplementation of 1.6 g taurine (without obvious adverse reactions) for 12 weeks significantly reduced office systolic ABP (by 7.2 mmHg), diastolic ABP (by 4.7 mmHg), and 24 h ambulatory ABP, with more significant effects in individuals with high normal ABP [93]. At this dose, taurine exerted its effects by increasing plasma H_2_S concentration and improving endothelium-dependent and independent vasodilation functions. The magnitude of ABP reduction was negatively correlated with plasma H_2_S and taurine levels, forming cross-species verification with the H_2_S pathway regulation mechanism in animal experiments. Meanwhile, this dose can be achieved through daily dietary fortification (e.g., 200 g salmon + 500 mL milk per day) or low-dose supplements, showing good clinical feasibility and safety [93].

Notably, the interventional effect of taurine is model-specific. In salt-resistant rats, even with a high-salt diet combined with taurine supplementation, there was no significant improvement in ABP, renal function, or renal tissue oxidative stress status, further confirming the targeted interventional value of taurine for SSH [19]. In summary, the 1.6 g/d dose for human intervention can be achieved through diet or low-dose supplements, which not only avoids the potential risks of supraphysiological doses but also exerts antihypertensive effects by regulating key pathological links of SSH, providing a scientific and feasible option for precise dietary intervention of SSH [93].

## 7. Conclusions

This review systematically integrates the complex pathological network of SSH, covering CNS regulation, subcellular stress, peripheral system dysfunction, and genetic-epigenetic modifications. It also comprehensively summarizes the interventional potential of dietary bioactive compounds, constructing a three-dimensional evaluation system encompassing pathological subtypes, compound mechanisms, and clinical evidence. This fills the current research gaps in insufficient multi-mechanism integration and systematic evaluation of dietary interventions in the field, providing a theoretical framework and practical reference for clinical dietary guidance and functional food development.

Among dietary bioactive compounds, taurine has the most complete evidence chain: preclinical studies using supraphysiological doses have clarified its multi-target regulatory effects on inflammation/oxidative stress, the RAAS, and ion channels. Clinical trials have confirmed that a human equivalent dose of 1.6 g/d (achievable through dietary fortification or low-dose supplements) can safely reduce ABP, completing cross-species verification of the H_2_S pathway mechanism. GABA also exhibits good translational prospects: doses in animal experiments are based on natural dietary sources, and the human equivalent dose of 0.3–2 g/d can be obtained through daily intake of fermented foods or specially cultivated fruits and vegetables. Studies on serum physiological levels indirectly confirm its safety.

Other compounds have obvious limitations in evidence translation: capsaicin and procyanidins show clear regulatory effects on pathways in CNS or RAAS in animal models, but their intervention doses are mostly supraphysiological or rely on non-dietary administration routes, lacking direct clinical evidence for SSH. For tea active components such as EGCG and black tea flavan-3-ols, clinical study results are inconsistent due to methodological heterogeneity and sample size issues, and the dose–effect relationship remains unclear. Caffeine has conflicting effects on ABP and no SSH-specific intervention evidence.

Current research still has significant limitations. Most compounds’ mechanisms are derived from animal experiments, where species differences and supraphysiological doses hinder result translation. Except for taurine, large-scale long-term clinical trials targeting SSH populations are lacking, and the synergistic effects between compounds and individualized intervention schemes remain unclarified. Future research should focus on core compounds to conduct SSH-specific clinical studies, optimize administration routes and dose design, and establish cross-species dose conversion standards. Multi-omics technologies should be applied to analyze the mechanisms of dietary bioactive compounds, facilitating their clinical translation in the precise intervention of SSH. This will provide more accurate and effective therapeutic options for clinical practice.

## Figures and Tables

**Table 1:** Regulatory factors among CNS in SSH: pathophysiological indicators and molecular mechanisms.

Influencing Factors	Origin	Pathophysiological Indicators	Molecular Mechanisms	References
SK	PVN	SNA ↑ ABP ↑ Neuronal firing ↑	SK channels ↓	[41,47,48]
Orexin system	PVN	ABP ↑ AVP ↑	OX1R ↑ CaMKII ↑	[49,50,51]
CO	PVN	SNA ↓; ABP ↓ NE ↓	COX2, IL-1β, IL-6, NOX2, and NOX4 ↓ HO-1 and Cu/Zn-SOD ↑	[52]
H_2_S	PVN	SNA ↓ABP ↓ HR ↓ NE ↓	NOX2, NOX4 and IL-1β ↓ H_2_S, CBS, IL-10 and Cu/Zn SOD ↑	[53]
Gαi2 protein	PVN Microglial cells	ABP ↑ Plasma noradrenaline ↑ Plasma renin activity ↑ Urinary angiotensinogen ↑	TNFα, IL-1β and IL-6 ↓ IL-10 ↑	[54]
NLRP3	PVN	ABP ↑ Plasma noradrenaline ↑ CD4+, CD8+ T cell, and CD8+ microglia ↑	CCL2, CXCR3, and VCAM-1 ↑ GAD67 ↓ TH ↑ GABA↓	[55]
TLR4	PVN	SNA ↑ ABP ↑	Myd88, NF-κB, PICs, IL-1β, IL-6, TNF-α, NOX2 and NOX4 ↑ SOD level ↓ TH ↑ GAD67 ↓	[56]
ROS	PVN	SNA ↑ ABP ↑	ACE, gp91(phox), gp47(phox) (subunits of NAD(P)H oxidase), AT1R, IL-1β, IL-6 ↑ IL-10 and Cu/Zn-SOD ↓	[57]
NF-κB	PVN	ABP ↑ NE ↑ EPI ↑	p-IKKβ, NF-κB p65 activity, Fra-LI activity (an indicator of chronic neuronal activation), NOX-4 (subunits of NAD(P)H oxidase), NLRP3, and IL-1β ↑ IL-10 ↓	[58]
TNF-α	PVN and cultured brain neurons from neonatal SD rats	ABP ↑	IL-1β, IL6, CCL5, CCL12, iNOS, and transcription factor NF-kB ↑	[59]

Abbreviations: ABP (arterial blood pressure), AVP (Arginine Vasopressin), ACE (Angiotensin-Converting Enzyme), AT1R (Angiotensin II type 1 receptor), CaMKII (Calcium/Calmodulin-Dependent Protein Kinase II), CBS (Cystathionine β-Synthase), CCL2 (C-C Motif Chemokine Ligand 2), CCL5 (C-C Motif Chemokine Ligand 5), CCL12 (C-C Motif Chemokine Ligand 12), CD4 (Cluster of Differentiation 4), CD8 (Cluster of Differentiation 8), CNS (central nervous system), CO (carbon monoxide), COX2 (Cyclooxygenase-2), CXCR3 (C-X-C Motif Chemokine Receptor 3), Cu/Zn-SOD (Copper/Zinc Superoxide Dismutase), EPI (Epinephrine), Galphai2 (G protein subunit alpha i2), Fra-LI (Fos-related antigen-like immunoreactivity), GAD67 (67 kDa Isoform of Glutamate Decarboxylase), GABA (gamma-aminobutyric acid), gp91(phox) (glycoprotein 91 (phagocyte oxidase)), gp47(phox) (glycoprotein 47 (phagocyte oxidase)), H_2_S (hydrogen sulfide), HO-1 (Heme Oxygenase-1), HR (heart rate), IKKβ (Inhibitor of Nuclear Factor Kappa-B Kinase Beta), IL-1β (Interleukin-1β), IL-6 (Interleukin-6), IL-10 (Interleukin-10), iNOS (Inducible Nitric Oxide Synthase), MyD88 (Myeloid Differentiation Factor 88), NE (Norepinephrine), NF-κB (Nuclear Factor Kappa B), NLRP3 (NOD-Like Receptor Family, Pyrin Domain Containing 3), NOX2 (NADPH Oxidase 2), NOX4 (NADPH Oxidase 4), OX1R (Orexin Type 1 Receptor), PICs (Pro-Inflammatory Cytokines), PVN (Paraventricular Nucleus of the Hypothalamus), p-IKKβ (phosphorylated inhibitor of nuclear factor kappa-B kinase beta), ROS (reactive oxygen species), SK (Small Conductance Ca^2+^-Activated K^+^ Channels), SNA (sympathetic nerve activity), SOD (Superoxide Dismutase), TNF-α (Tumor Necrosis Factor-α), TLR4 (Toll-Like Receptor 4), TH (Tyrosine Hydroxylase), VCAM-1 (Vascular Cell Adhesion Molecule-1), gp91(phox) (Glycoprotein 91 Phagocyte Oxidase), gp47(phox) (Glycoprotein 47 Phagocyte Oxidase), ACE (Angiotensin-Converting Enzyme), AT1R (Angiotensin II Type 1 Receptor), Gαi2 (G Alpha i2 Protein), SD rats (Sprague Dawley rats). ↑ indicates increased; ↓ indicates decreased.

**Table 2:** Natural compounds: sources, target organs, arterial blood pressure-regulating mechanisms, and dosages.

Chemical Compound	Source	Human Dosage/ Animal Dosage	Target Organ	Pathophysiological Indicators	Main Mechanism of Action	References
Capsaicin	Chili	---/ DSS rats: PVN infusion	PVN	Thickness of ventricular walls and shrunken heart chambers, ↓ ANP and BNP ↓	NOX2, iNOS, NOX4, and p-IKKβ ↓ Nrf2 and HO-1 ↑ p-PI3K and p-AKT ↓ p-AMPK ↑	[91,92]
ALA	Spinach, broccoli, and animal livers	---/ Via gastric perfusion (60 mg/kg for 9 weeks)	PVN	SNA ↑ ABP ↑	ACE, gp91(phox), gp47(phox) (subunits of NAD(P)H oxidase), AT1R, IL-1β, IL-6 ↑ IL-10 and Cu/Zn-SOD ↓	[57]
Taurine	Meat, seafood, dairy, and other foods	---/ DSS rats: 2%~3% added to drinking water (4~6 weeks, equivalent to 10~15 g/d in humans, supraphysiological dose)	Renal tissue	ABP ↓ CDO1 and CSAD ↑	SOD1, SOD2 ↑ Renin, gp91phox, p22phox, and p47phox ↓	[18,19,93]
GABA	Fermented foods and specific plants	0.3–2 g/d (dietary equivalent dose)/Hypertensive mice: GABA salt (based on dietary salt intake ratio)	Vascular function	ABP ↓ EC dysfunction ↓	GABAB receptor and eNOS phosphorylation ↑ E-selectin, ICAM-1, VCAM-1 and EC ↓ Endothelin-1 levels ↓	[17,94,95,96]
Procyanidins	Mainly derived from plant tissues such as grape seeds and blueberries	0–2.5 mg/kg body weight/day (acceptable daily intake, ADI)/high-salt-induced hypertensive rats: 50, 100, 200 mg/kg	Serum RAAS	ABP and heart rate ↓	Serum ACE and plasma aldosterone ↓	[16]
EGCG	Tea	No clear SSH-specific dose	Renal tissue	ABP ↓ 24 h urine protein levels, creatinine clearance, renal fibrosis ↓	Malondialdehyde levels, the number of infiltrated macrophages and T cells ↓	[15]

“---”: No relevant clinical evidence or human reference dose. Abbreviations: ABP (arterial blood pressure); ACE (Angiotensin-Converting Enzyme); ADI (Acceptable Daily Intake), ALA (Alpha Lipoic Acid); ANP (Atrial Natriuretic Peptide); AT1R (Angiotensin II type 1 receptor), BNP (B-Type Natriuretic Peptide); CDO1 (Cysteine Dioxygenase Type 1); CSAD (Cysteine Sulfinic Acid Decarboxylase); Cu/Zn-SOD (Copper/Zinc Superoxide Dismutase); EC (endothelial cell); eNOS (Endothelial Nitric Oxide Synthase); EGCG (Epigallocatechin Gallate); GABA (gamma-aminobutyric acid); GABAB (gamma-aminobutyric acid type B); gp91(phox) (glycoprotein 91 (phagocyte oxidase)), gp47(phox) (glycoprotein 47 (phagocyte oxidase)), HO-1 (Heme Oxygenase-1); ICAM-1 (Intercellular Adhesion Molecule-1); IL-1β (Interleukin-1β); IL-6 (Interleukin-6); IL-10 (Interleukin-10); iNOS (Inducible Nitric Oxide Synthase); IKKβ (Inhibitor of Nuclear Factor Kappa-B Kinase Beta); NAD(P)H (Nicotinamide Adenine Dinucleotide (Phosphate)); NOX2 (NADPH Oxidase 2); NOX4 (NADPH Oxidase 4); Nrf2 (Nuclear Factor Erythroid 2-Related Factor 2); p-AMPK (Phosphorylated Adenosine Monophosphate-Activated Protein Kinase); p-AKT (Phosphorylated Protein Kinase-B); p-IKKβ (Phosphorylated Inhibitor of Nuclear Factor Kappa-B Kinase Beta); p-PI3K (Phosphorylated Phosphatidylinositol 3-Kinase); PVN (Paraventricular Nucleus of the Hypothalamus); p22phox (cytochrome b-245 alpha polypeptide), p47phox (neutrophil cytosolic factor 1), RAAS (renin–angiotensin–aldosterone system); SNA (sympathetic nerve activity); SOD1 (Superoxide Dismutase 1); SOD2 (Superoxide Dismutase 2); SSH (salt-sensitive hypertension); VCAM-1 (Vascular Cell Adhesion Molecule-1); VSMC (vascular smooth muscle cell). ↑ indicates increased; ↓ indicates decreased.

## Data Availability

The original contributions presented in this study are included in the article. Further inquiries can be directed to the corresponding author(s).
